# Late Local and Distant Recurrence of Apparently Benign Paraganglioma

**DOI:** 10.7759/cureus.29874

**Published:** 2022-10-03

**Authors:** Brijesh K Singh, Toshib G A, Hemanga K Bhattacharjee, Rajinder Parshad, Nishikant A Damle

**Affiliations:** 1 Surgery, All India Institute of Medical Sciences, New Delhi, IND; 2 Nuclear Medicine, All India Institute of Medical Sciences, New Delhi, IND

**Keywords:** tumor recurrence, video-assisted thoracoscopic surgery (vats), hypertensive crisis, nephrectomy, paraganglioma

## Abstract

Paraganglioma-pheochromocytoma (PPGLs) are relatively rare catecholamine-secreting tumors of chromaffin origin. Due to the sympathetic effects of catecholamine excess, their presentation may range from non-specific symptoms to dangerous hypertensive crises. We present the case of a 36-year-old lady with recurrent paraganglioma (PGL) who presented in emergency with hypertensive crisis. She had a history of surgery for left-sided PGL 18 years earlier. Imaging showed local recurrence with pulmonary metastases and blood biochemistry showed raised urinary metanephrines. In view of her poor general condition, we undertook a staged surgical approach for management. She first underwent en-bloc excision of recurrent PGL with left nephrectomy. Nine weeks later, she underwent a pulmonary metastasectomy. This staged surgical approach resulted in the stabilization of blood pressure and normalization of urinary catecholamine. Although most of these tumors are indolent by nature, this case highlights the metastatic potential of apparently benign PGL. This case explores the possibility of a staged surgical approach in a high-risk patient and emphasizes the need for long-term follow-up in these cases.

## Introduction

Paraganglioma (PGL) and pheochromocytoma (PCC) are neuroendocrine tumors of chromaffin origin. PGL arises from extra-adrenal paraganglia while PCC originates from the adrenal medulla [1]. They are collectively referred to as pheochromocytoma-paraganglioma (PPGL). Most of the cases are apparently benign and complete surgical resection results in tumor-free outcomes. However, PPGL has metastatic potential requiring long-term follow-up [2].

Presentation of PPGL often gets delayed due to the indolent nature of the disease and poor awareness among the patients. Even apparently benign PPGL may recur decades after initial diagnosis. Patients with recurrent disease manifest late in a debilitating condition. Such recurrences are difficult to manage. We are describing a case of PGL with recurrence 18 years after initial treatment. We have highlighted the staged surgical management of local and distant recurrence in the present report.

## Case presentation

A 36-year-old female presented in the emergency department for the evaluation of worsening headache and altered sensorium. On examination, her systolic blood pressure (BP) was 200 mmHg, Glasgow coma scale (GCS) was 14/15 with right-sided hemiparesis. There was no history of seizures, fever, ear discharge, head trauma, and bladder or bowel involvement. Investigations revealed deranged creatinine of 2.5 mg/dL (normal range: 0.5 - 1 mg/dL). Magnetic resonance imaging (MRI) brain was suggestive of multiple infracts.

Significant past clinical details included a history of surgery for left-sided PGL 18 years back. At that time, she had decreased vision (grade IV hypertensive retinopathy), resistant hypertension, and elevated 24-hours urinary norepinephrine. A 5×7 cm^2^ encapsulated mass abutting the inferior pole of the left kidney was excised completely. Histopathology (HPE) of the operated specimen was compatible with PGL.

**Figure 1:**
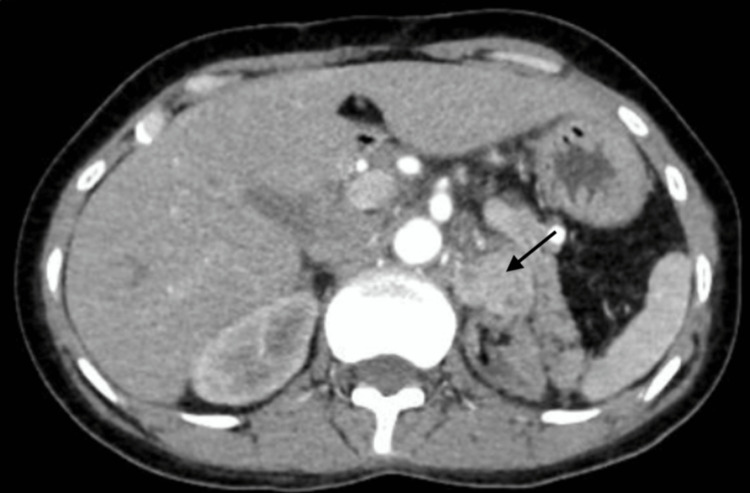
Contrast-enhanced CT scan of the abdomen Showing a left paraaortic mass (arrow) encasing left renal vessels

During the present hospitalization, after stabilization of the BP with intravenous nitro-glycerine, imaging studies were performed. MRI abdomen showed left paraaortic mass suggestive of recurrent PGL with the uptake of metaiodobenzylguanidine (MIBG). Contrast-enhanced CT scan of the abdomen revealed a 2.6×2.7 cm^2^ mass arising anterior to the left kidney with a few enlarged lymph nodes completely encasing the left renal artery and vein (Figure 1) with left renal atrophy measuring 5.3×2.2 cm^2^.

**Figure 2:**
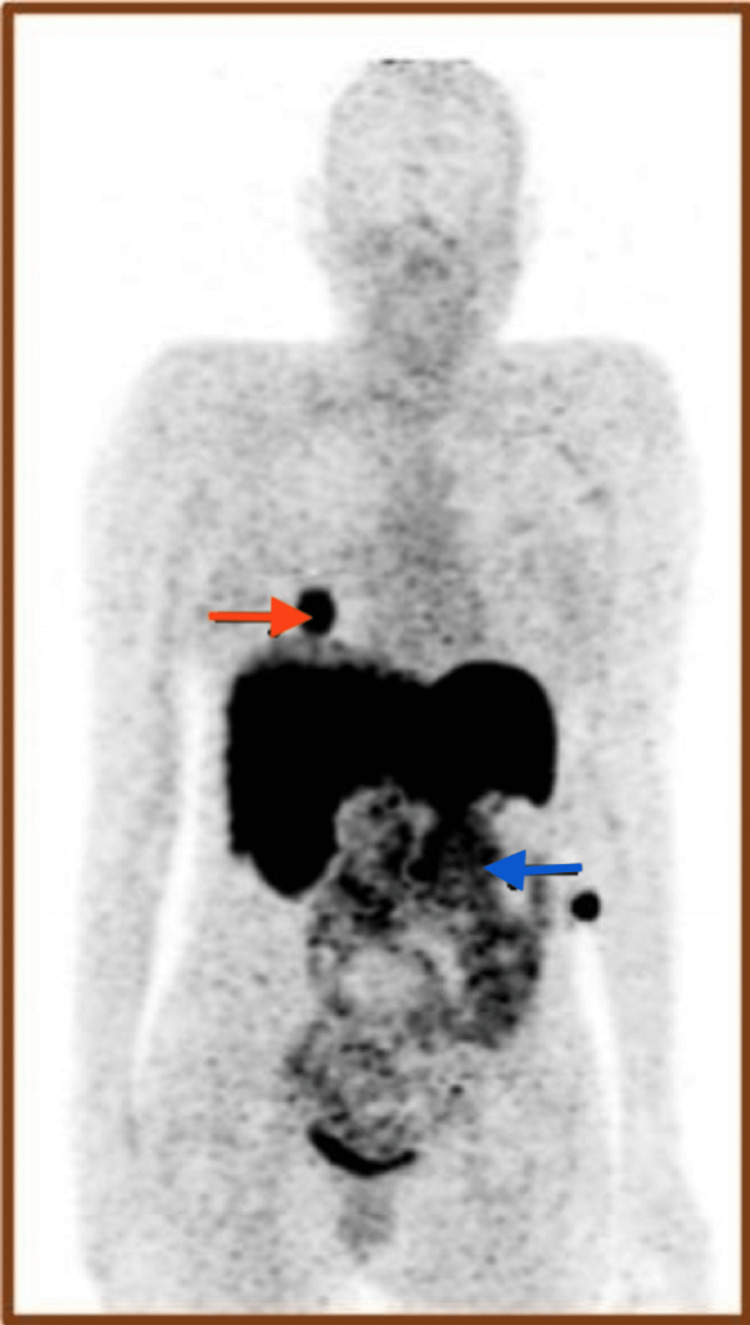
68Ga-DOTANOC whole-body PET/CT Showing increased radiotracer uptake in the left paraaortic region (blue arrow) and a metastatic nodule in the right lung lower lobe (red arrow) before surgery

A 68Ga-DOTANAC whole-body PET/CT scan revealed radiotracer uptake in the left paraaortic region of the abdomen, and right pulmonary lower lobe (Figure 2) suggestive of local recurrence and lung metastasis, respectively.

**Figure 3:**
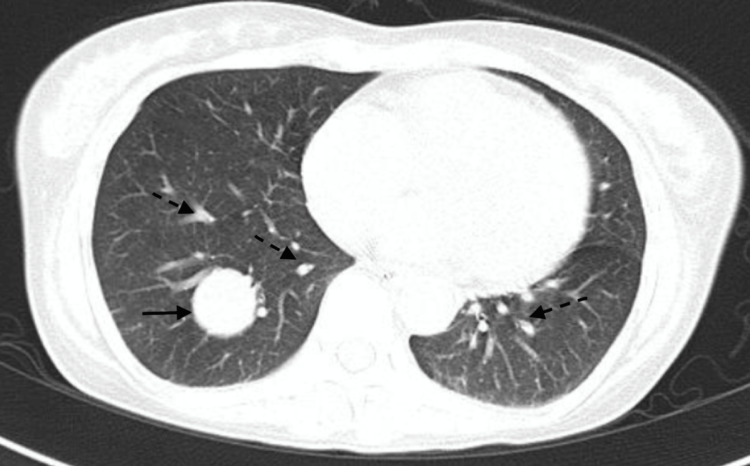
CT thorax Showing metastatic nodule (solid arrow) in the right lower lobe with multiple suspicious nodules (broken arrows) in both lungs

CT thorax revealed a hypodense 2.5×2.6 cm^2^ nodule in the right lower lobe, 0.6×0.5 cm^2^ nodule in the left lower lobe, and multiple centrilobular as well acinar nodules in the left lung suggestive of metastasis (Figure 3). MRI brain was suggestive of hypertensive changes. Free urinary metanephrines were elevated (438 μg/day, normal 25-312 μg/day). A diethylenetriamine pentaacetate (DTPA) scan for renal function revealed a poorly functioning left kidney and preserved right kidney function (split function: left 7.5%, right 92.5%). A detailed clinical assessment revealed no syndromic association.

**Figure 4:**
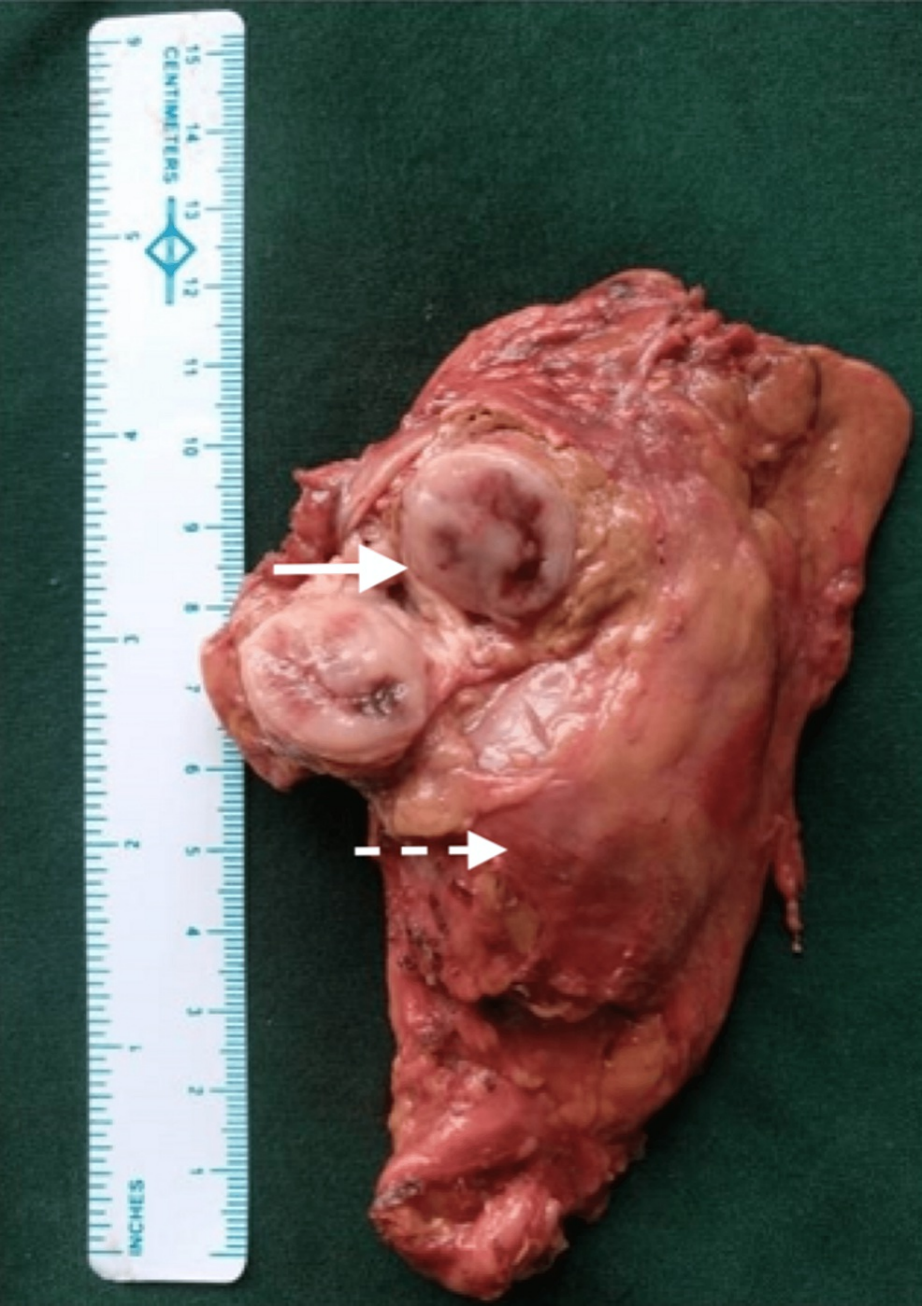
Abdominal local site resection specimen Specimen of en-bloc excision of paraganglioma (solid arrow) with left nephrectomy (broken arrow) and left adrenalectomy

In view of her poor general condition and multiple lung metastases, we planned staged surgery with initial resection of abdominal recurrence. The patient underwent an exploratory laparotomy, which showed a firm paraaortic mass at the left renal hilum adherent to renal vessels with enlarged paraaortic lymph nodes. En-bloc excision of the mass along with the left adrenal gland, left kidney, and enlarged lymph nodes was done (Figure 4).

**Figure 5:**
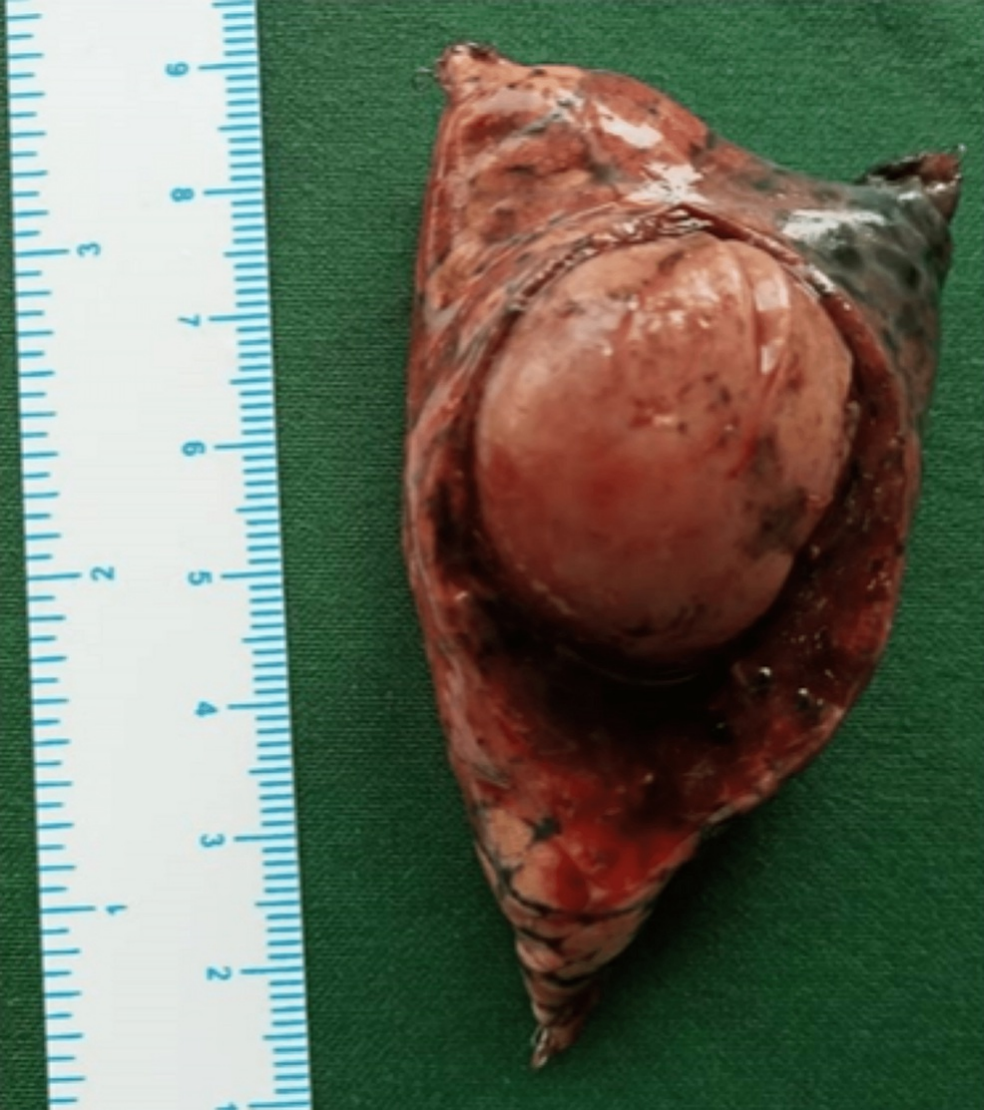
Lung metastatic nodule resection specimen Specimen of wedge resection of right lung lower lobe with metastatic nodule by video-assisted thoracoscopic surgery.

Wedge resection of the right pulmonary nodule was done by video-assisted thoracic surgery (VATS) nine weeks after laparotomy (Figure 5). Histopathology of abdominal mass was suggestive of PGL, immunopositive for synaptophysin, and mitotic count of two per 10 high power field. The adrenal gland was histologically unremarkable and the kidney showed features of chronic nephritis. Histopathology of the resected lung specimen was also compatible with metastatic PGL.

**Figure 6:**
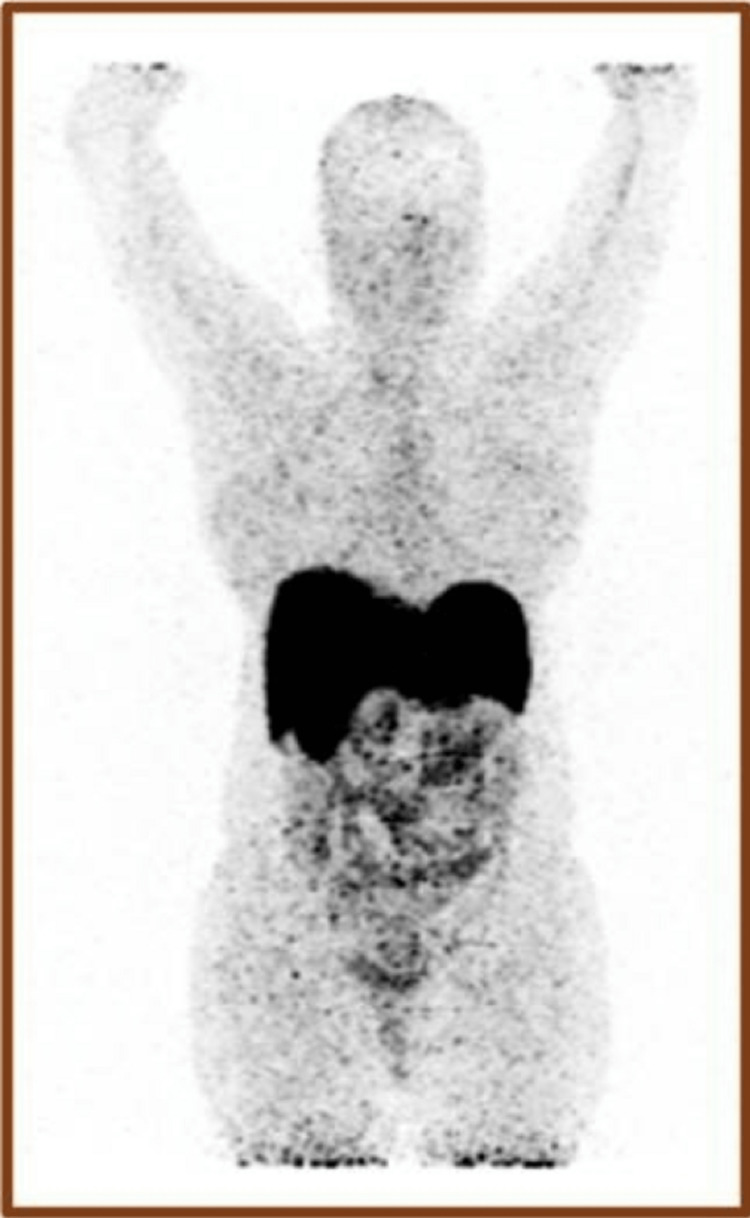
Follow-up 68Ga-DOTANOC whole-body PET/CT scan Showing no abnormal uptake

The patient made an uneventful recovery after two surgeries. A repeat 68Ga-DOTANOC whole-body PET/CT at three months after pulmonary nodule resection showed no uptake in the left paraaortic and pulmonary areas (Figure 6). No adjuvant chemotherapy was offered. Her blood pressure was maintained on a single antihypertensive drug, and urinary catecholamine levels were normal at 18 months of follow-up.

## Discussion

The malignant potential of PPGL is a controversial issue. Metastatic disease is difficult to predict by clinical, histological, or biochemical features. The diagnosis of malignancy is based on local invasion or the presence of metastasis in non-chromaffin tissue [1]. The most common sites of metastasis include regional lymph nodes, lungs, bones, and liver [3]. The time of recurrence after initial diagnosis may vary from six months to 17 years after surgery [3,4]. The present report describes a case of recurrent PGL 18 years after initial diagnosis along with lung metastasis. Long-term follow-up of PGL patients with an apparently benign disease is important due to their potential for delayed recurrences.

Extra-adrenal masses (paraganglioma) and PPGL associated with inherited syndromes are more likely to recur [4]. PPGL with syndromic association tends to develop at a younger age [5,6]. They are more often bilateral, multifocal, and recurrent compared to sporadic diseases [5,6]. Clinical features predicting the malignant potential of PPGL include large tumor size (>5.5 cm) and persistence of postoperative arterial hypertension [7]. Histological parameters predicting increased risk of malignancy include the Pheochromocytoma of the Adrenal Gland Scaled Score (PASS), Ki-67 index, HSP 90 (heat-shock protein 90), and activator of transcription 3 (STAT 3) [8,9]. However, PASS is applicable only for PCC, and the role of all these markers in predicting malignancy is controversial [9]. The occurrence of large extra-adrenal chromaffin tumors at the young age of 18 years predicted a high probability of recurrence in the present case.

Surgical resection is the treatment of choice for localized PPGL [10]. Debulking surgery is not curative for metastatic disease, however, it helps reduce the tumor burden and catecholaminergic effects. Patients with metastatic disease and having good performance status without evidence of multiple metastases, and those with a solitary target tumor are candidates for surgery [11]. In unresectable cases of PPGL, I-131 labeled MIBG therapy is recommended but complete remission rarely occurs [12,13]. Treatment with radioactive somatostatin analogs, such as Lutetium-177 DOTATATE or Yttrium-90 DOTATATE, increases overall and event-free survival compared to I-131 MIBG in these cases [2]. In the present case, the majority of the tumor burden was localized in the retroperitoneum, which was resected first followed by right pulmonary nodule excision. Due to the poor general condition of the patient, we chose to undertake a staged approach. Upfront excision of the abdominal mass reduced the tumor burden, thus helping control blood pressure and improving her effort tolerance for lung surgery.

The major debulking surgery in our patient may have resulted in the spontaneous resolution of smaller lung metastases. Few reports highlight the spontaneous regression of PPGL. Hammer et al. reported the spontaneous disappearance of the left carotid body tumor eight years after the excision of contralateral PGL [14]. Chang et al. and Nakajima et al. described spontaneous regression followed by recurrence of retroperitoneal PGL [15,16]. The possible mechanisms leading to spontaneous regression in these cases include genetic instability (telomerase inhibition), apoptosis, changes in tumor blood supply, loss of growth-promoting factors after excision of large lesions, and hemorrhage followed by resorption [14].

PPGLs are rare tumors with a high probability of recurrence even decades after the initial diagnosis. The Endocrine Society Clinical Practice Guideline (ESCPG) advises lifelong follow-up while the European Society of Endocrinology (ESE) suggests a 10-year follow-up for all operated cases of PPGL [17,18]. ESE also endorses lifelong follow-up for high-risk patients with disease onset at a younger age, genetic disease, large tumor, and/or PGL. Follow-up activities suggested include biochemical tests yearly and imaging every one to two years [17,18]. Our patient was a high-risk case requiring intense follow-up. However, her poor socio-economic condition precluded regular follow-up, leading to a delayed diagnosis of the recurrence. A detailed genetic evaluation could have given us more information about her disease, however, could not be performed due to financial constraints.

## Conclusions

The present case emphasizes the need for long-term regular follow-up of all cases of PPGL. Patients should be educated regarding the possible delayed recurrence of an apparently benign PPGL. In patients with poor performance status and extensive disease, staged surgery is an excellent option. Debulking surgery may lead to spontaneous resolution of small metastatic nodules and improve long-term survival.
